# Supplementary material for: Association between oral microbiome and seven types of cancers in East Asian population: a two-sample Mendelian randomization analysis
Source: Front Mol Biosci. 2023 Nov 21;10:1327893. doi: 10.3389/fmolb.2023.1327893 (PMC10702768; doi:10.3389/fmolb.2023.1327893)
Supplement: Supplementary file 1 [file DataSheet1.ZIP › Supplementary Materials/MR plots for tongue/tongue═╝/Colorectal cancer/pheno.2764_to_colorectal cancer_forest.pdf]

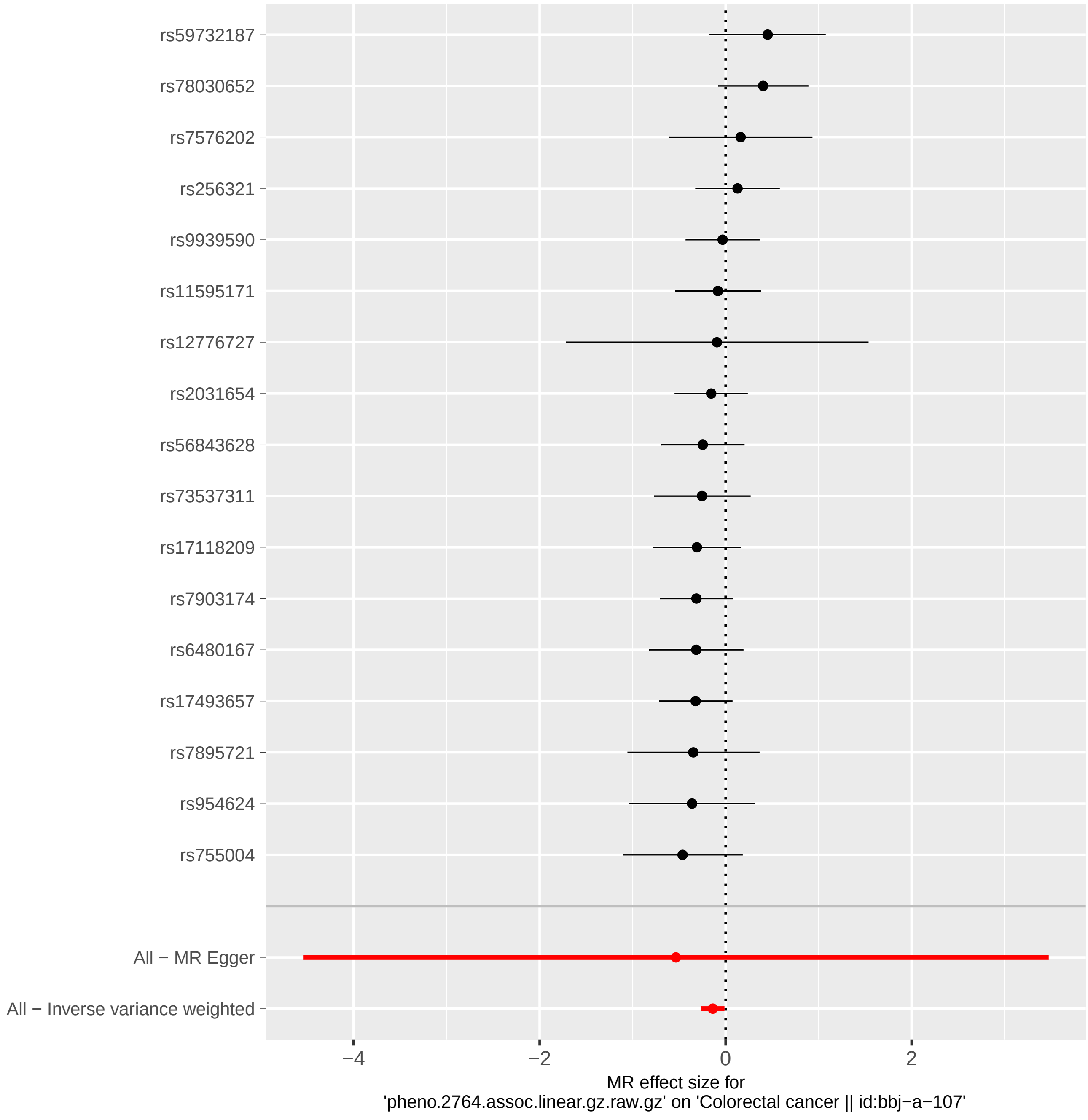
rs59732187
rs78030652
rs7576202
rs256321
rs9939590
rs11595171
rs12776727
rs2031654
rs56843628
rs73537311
rs17118209
rs7903174
rs6480167
rs17493657
rs7895721
rs954624
rs755004
All − MR Egger
All − Inverse variance weighted
−4
−2
0
2
MR effect size for
'pheno.2764.assoc.linear.gz.raw.gz' on 'Colorectal cancer || id:bbj−a−107'
